# Subclinical left ventricular diastolic dysfunction in adults with overweight or obesity and preserved ejection fraction

**DOI:** 10.1016/j.obpill.2026.100287

**Published:** 2026-06-18

**Authors:** Nikita Chanana, Poonam Ashok Kamath, H. Manjunatha Hande, Kanhai Lalani

**Affiliations:** a Department of General Medicine, Kasturba Medical College, Manipal Academy of Higher Education, Manipal, India; b Department of Cardiology, Kasturba Medical College, Manipal Academy of Higher Education, Manipal, India

**Keywords:** Adiposity, Diastolic dysfunction, Echocardiography, Heart failure with preserved ejection fraction, Obesity, Overweight

## Abstract

**Background:**

Excess adiposity exerts haemodynamic, metabolic, and inflammatory effects on the myocardium that can produce left ventricular diastolic dysfunction (LVDD) before systolic function is impaired. South Asian adults are susceptible at lower body mass index (BMI) thresholds, yet evidence on subclinical LVDD in otherwise healthy adults with overweight or obesity is limited.

**Methods:**

Tertiary care hospital-based, cross-sectional study (June 2024–February 2026). 72 adults were enrolled aged 18–65 years with BMI ≥23 kg/m^2^ (Asian cut-offs) and preserved left ventricular ejection fraction (LVEF) > 50%, with no previously diagnosed cardiovascular disease or other established causes of diastolic dysfunction after excluding hypertension, diabetes, coronary, valvular, renal, thyroid, and pulmonary disease, sleep apnoea, smoking, and alcohol use. All underwent 2D transthoracic echocardiography classified per 2016 ASE/EACVI recommendations.

**Results:**

Grade I LVDD was present in 29/72 participants (40.3%); none had grade 2 or grade 3 dysfunction. Affected adults were older (between 51 and 60 years of age) with higher HbA1c (5.52 vs 5.27%), LDL cholesterol (138.2 vs 110.9 mg/dL), and total cholesterol (205.9 vs 173.9 mg/dL). Mean BMI did not differ (27.9 vs 28.9 kg/m^2^), although BMI ≥25 kg/m^2^ as a binary indicator was associated with LVDD. E/A (0.76 vs 1.33) and septal e′ (0.09 vs 0.11 m/s) were reduced; E/e′ trended higher (8.29 vs 7.61). LVEF was preserved (∼64.7%) in both groups. No safety events occurred.

**Conclusion:**

Grade I left ventricular diastolic dysfunction was identified in 40.3% of adults with overweight or obesity and preserved systolic function who had no previously diagnosed cardiovascular disease or other established causes of diastolic dysfunction. Older age, higher HbA1c, and adverse lipid profiles were associated with LVDD, whereas BMI as a continuous variable was not. These findings suggest that subclinical diastolic abnormalities may be common in this population and support the need for prospective studies to determine their long-term clinical significance and relationship to future cardiovascular outcomes.

## Highlights

• Among adults with overweight or obesity but no other cardiac comorbidities, 40.3% had Grade I left ventricular diastolic dysfunction despite preserved ejection fraction.
• Older age, higher HbA1c, and higher LDL and total cholesterol levels were significantly associated with subclinical diastolic dysfunction.
• Tissue Doppler indices (E/A and septal e′) were markedly reduced in affected adults, whereas E/e′ remained <14, consistent with early disease.
• BMI as a continuous variable did not discriminate between groups, suggesting that echocardiographic screening should not be reserved for the highest BMI strata.
• Routine integration of diastolic assessment, lipid optimisation, and weight management may help avert progression to heart failure with preserved ejection fraction.

## Graphical abstract

**Figure:**
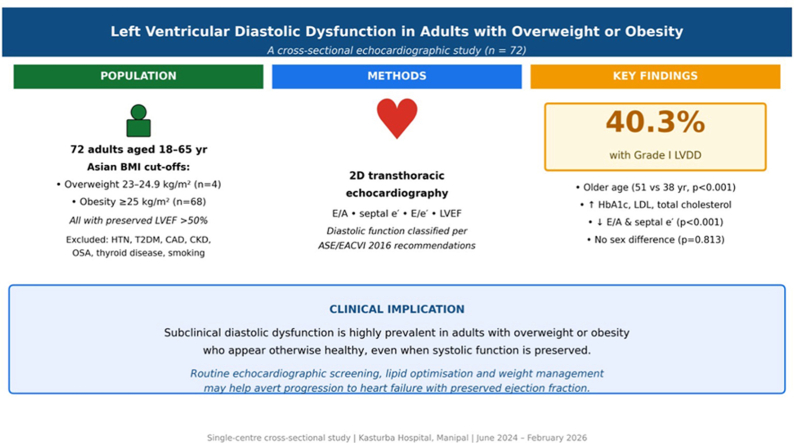

## 1 Introduction

Overweight and obesity have emerged as one of the most consequential health burdens in current times. The World Health Organization defines overweight as a body mass index (BMI) of 25 kg/m^2^ or greater and obesity as a BMI of 30 kg/m^2^ or greater, with lower thresholds (≥23 kg/m^2^ and ≥25 kg/m^2^, respectively) endorsed for Asian populations because cardiometabolic risk emerges at lower BMI [1,2]. Globally, more than 1.9 billion adults are now classified as overweight and over 650 million as obese [1]. India is undergoing a particularly rapid epidemiological transition, with sedentary lifestyles, urbanisation, and energy-dense diets driving increases in adiposity across all socioeconomic strata [3,4].

The cardiovascular consequences of this transition extend well beyond conventional risk factors. Excess adiposity independently increases cardiovascular risk through chronic haemodynamic overload, neurohormonal activation, systemic low-grade inflammation, epicardial adipose tissue secretion of pro-inflammatory cytokines, and intramyocardial lipid accumulation [[5], [6], [7], [8], [9]]. These mechanisms converge on the diastolic phase of the cardiac cycle. Delayed myocardial relaxation, increased interstitial fibrosis, and reduced ventricular compliance produce elevated filling pressures even when systolic ejection is preserved, providing a mechanistic link to heart failure with preserved ejection fraction (HFpEF) [3,10].

Echocardiography remains the most accessible and reproducible tool for the early detection of left ventricular diastolic dysfunction (LVDD). The 2016 American Society of Echocardiography and European Association of Cardiovascular Imaging (ASE/EACVI) recommendations integrate transmitral Doppler indices (E/A ratio), tissue Doppler velocities (e′), and the E/e′ ratio to grade diastolic function [11]. Studies in Western and Asian cohorts have consistently shown reduced e′ velocity, elevated E/e′, and increased left atrial volume index in adults with overweight or obesity compared with lean controls [[12], [13], [14], [15], [16]]. However, several considerations warrant additional region-specific evidence. South Asian populations exhibit greater visceral adiposity and metabolic risk at lower BMI than other ethnic groups [7], BMI as a continuous variable may imperfectly capture cardiac vulnerability within an already overweight cohort, and most earlier studies were enriched with adults who also had hypertension or diabetes, making it difficult to isolate the contribution of adiposity itself [[17], [18], [19]].

Therefore a single-centre, cross-sectional study was conducted at a tertiary care hospital in southern India to estimate the prevalence of LVDD in adults with overweight or obesity, preserved LVEF, and no other established causes of diastolic dysfunction, and to characterise its association with demographic, anthropometric, glycaemic, and lipid variables. It was hypothesised that subclinical LVDD would be common in this group and that its detection would be independently associated with advancing age and with metabolic abnormalities that fall short of conventional disease thresholds.

This study aimed to assess the prevalence and correlates of left ventricular diastolic dysfunction in adults with overweight or obesity with no previously diagnosed cardiovascular disease or other established causes of diastolic dysfunction. Primary objective was to estimate the proportion of left ventricular diastolic dysfunction in adults with overweight and obesity and as a secondary objective we determined the association between BMI and other clinical and biochemical variables with left ventricular diastolic dysfunction.

## 2 Materials and methods

### 2.1 Study design and setting

This was a single-centre, hospital-based, cross-sectional observational study. The investigation was conducted in the Department of General Medicine at Kasturba Hospital, Manipal Academy of Higher Education, Manipal, Karnataka, India, between June 2024 and February 2026. Patients coming to the hospital in department of cardiology and general medicine were taken for the study.

### 2.2 Participants and eligibility criteria

Adults aged 18–65 years with BMI ≥23 kg/m^2^ (Asian cut-offs) and preserved left ventricular ejection fraction (LVEF >50%) on screening echocardiography were eligible. Participants were classified as having overweight (BMI 23.0–24.9 kg/m^2^) or obesity (BMI ≥25 kg/m^2^) per the consensus guidelines for Asian populations [2,7]. Adults with any of the following conditions were excluded to isolate the contribution of adiposity to diastolic function: hypertension, type 2 diabetes mellitus, coronary artery disease, valvular heart disease, cardiomyopathy, chronic kidney disease, obstructive sleep apnoea, thyroid disorders, severe anaemia (haemoglobin <8 g/dL), acute infections, chronic lung disease, HIV infection, current smoking, regular alcohol use, and use of drugs known to influence diastolic function.

### 2.3 Sampling and sample size

The sample size was calculated using the formula for estimating a single proportion (Lemeshow et al., 1990), based on a previously reported prevalence of diastolic dysfunction of 24.6% in adults with obesity [17] which was 72.

### 2.4 Clinical and biochemical assessment

All participants underwent a structured clinical evaluation including detailed history and review of medical records, standardized anthropometry (height to the nearest 0.1 cm using a stadiometer; weight to the nearest 0.1 kg using a calibrated digital scale; BMI calculated as weight in kilograms divided by height in metres squared), and resting brachial blood pressure measured after at least 5 min of rest. Fasting blood samples were obtained for haematological and biochemical investigations including complete blood count, glycated haemoglobin (HbA1c), fasting lipid profile (total cholesterol, low-density lipoprotein cholesterol, high-density lipoprotein cholesterol, triglycerides), liver and renal function tests, and thyroid profile. Lipid risk categories followed Adult Treatment Panel III thresholds.

### 2.5 Echocardiographic evaluation

Two-dimensional transthoracic echocardiography was performed by trained personnel using a standard cardiac transducer, in accordance with 2016 ASE/EACVI recommendations [11].

Parameters recorded included transmitral pulsed-wave Doppler early (E) and atrial (A) velocities and their ratio (E/A), tissue Doppler imaging of septal e′ velocity at the mitral annulus, the E/e′ ratio, peak tricuspid regurgitation (TR) velocity, and biplane Simpson's LVEF. Diastolic function was classified as normal or Grade I dysfunction (impaired relaxation) by integrating these parameters per the 2016 algorithm [11]. Equipment was calibrated according to the manufacturer's recommendations during the study period.

### 2.6 Statistical analysis

Data were entered into Microsoft Excel and analysed using IBM SPSS Statistics version 23 (IBM Corporation, Armonk, NY, USA). Continuous variables were summarised as mean ± standard deviation or median (interquartile range), and categorical variables as frequency and percentage. The Shapiro-Wilk test assessed normality. Between-group comparisons used the independent-sample *t*-test for normally distributed continuous variables and the Mann-Whitney *U* test (Wilcoxon-Mann-Whitney) when the distribution was non-normal. Univariable logistic regression was used to estimate odds ratios for Grade I LVDD with each candidate predictor; given the modest sample size, no multivariable model was fitted. A two-sided p < 0.05 was taken as statistically significant.

### 2.7 Ethics

The study protocol was approved by the Institutional Ethics Committee of Kasturba Medical College and Kasturba Hospital, Manipal Academy of Higher Education, Manipal [IEC approval number: IEC2: 205/2024] and was registered with the Clinical Trials Registry of India [CTRI registration number: CTRI/2024/08/073115]. The study was conducted in accordance with the Declaration of Helsinki of the World Medical Association. All participants provided written informed consent in a language they understood, after the purpose, procedures, and rights of withdrawal had been explained. Participant data were de-identified and stored securely with restricted access. No clinical care decisions were altered by participation in the study.

## 3 Results

### 3.1 Participant characteristics

Seventy-two adults with overweight or obesity were enrolled and completed echocardiographic evaluation. Study comprised 46 men (63.9%) and 26 women (36.1%) with a mean age of 43.4 ± 12.2 years. Per Asian BMI cut-offs, 4 participants (5.6%) had overweight (BMI 23.0–24.9 kg/m^2^) and 68 (94.4%) had obesity (BMI ≥25 kg/m^2^); the mean BMI of the cohort was 28.5 ± 3.7 kg/m^2^. The mean LVEF was 64.7 ± 2.2%, confirming preserved systolic function across the sample. Detailed baseline characteristics, stratified by diastolic function status, are presented in Table 1.

**Table 1:** Demographic, anthropometric, clinical characteristics, biochemical and lipid profile parameters of study participants (n = 72), stratified by left ventricular diastolic function status.

Parameter	Overall (n = 72)	Normal diastolic function (n = 43)	Grade I LVDD (n = 29)
Age, years, mean ± SD	43.4 ± 12.2	37.9 ± 10.6	51.5 ± 9.8
Age group, n (%)			
20–29	11 (15.3)	10 (23.3)	1 (3.4)
30–39	20 (27.8)	16 (37.2)	4 (13.8)
40–49	13 (18.1)	10 (23.3)	3 (10.3)
50–59	21 (29.2)	5 (11.6)	16 (55.2)
60–69	7 (9.7)	2 (4.7)	5 (17.2)
Sex, n (%)			
Men	46 (63.9)	27 (62.8)	19 (65.5)
Women	26 (36.1)	16 (37.2)	10 (34.5)
Height, cm, mean ± SD	166.3 ± 9.0	166.4 ± 9.2	166.1 ± 8.9
Weight, kg, mean ± SD	79.0 ± 14.7	80.2 ± 15.5	77.2 ± 13.4
BMI, kg/m^2^, mean ± SD	28.5 ± 3.7	28.9 ± 4.1	27.9 ± 3.1
BMI category, n (%)			
Overweight (23.0–24.9 kg/m^2^)	4 (5.6)	0 (0.0)	4 (13.8)
Obesity (≥25 kg/m^2^)	68 (94.4)	43 (100.0)	25 (86.2)
Pulse rate, bpm, mean ± SD	76.1 ± 11.2	74.9 ± 12.6	77.7 ± 8.7
Systolic BP, mmHg, mean ± SD	120.7 ± 10.3	118.5 ± 10.2	124.0 ± 9.6
Diastolic BP, mmHg, mean ± SD	78.1 ± 6.6	78.0 ± 6.6	78.3 ± 6.8
Haemoglobin, g/dL	14.2 ± 1.4	14.0 ± 1.4	14.4 ± 1.3
Total leukocyte count, × 10^3^/μL	7.2 ± 1.5	7.2 ± 1.5	7.2 ± 1.6
Platelet count, × 10^3^/μL	266.5 ± 60.2	272.6 ± 61.0	257.5 ± 58.9
HbA1c, %	5.38 ± 0.39	5.27 ± 0.36	5.52 ± 0.39
HbA1c category, n (%)			
<5.7%	53 (73.6)	35 (81.4)	18 (62.1)
5.7–6.4%	19 (26.4)	8 (18.6)	11 (37.9)
HDL cholesterol, mg/dL	46.2 ± 11.9	46.2 ± 12.4	46.1 ± 11.3
LDL cholesterol, mg/dL	121.9 ± 27.0	110.9 ± 18.2	138.2 ± 29.9
LDL category, n (%)			
<100 mg/dL	14 (19.4)	11 (25.6)	3 (10.3)
100–129 mg/dL	38 (52.8)	29 (67.4)	9 (31.0)
130–189 mg/dL	17 (23.6)	3 (7.0)	14 (48.3)
≥190 mg/dL	3 (4.2)	0 (0.0)	3 (10.3)
Triglycerides, mg/dL	117.9 ± 46.4	107.0 ± 35.3	134.0 ± 56.0
Total cholesterol, mg/dL	186.8 ± 31.3	173.9 ± 20.6	205.9 ± 34.8
Total cholesterol category, n (%)			
<200 mg/dL	53 (73.6)	39 (90.7)	14 (48.3)
200–239 mg/dL	14 (19.4)	4 (9.3)	10 (34.5)
≥240 mg/dL	5 (6.9)	0 (0.0)	5 (17.2)
Creatinine, mg/dL	0.87 ± 0.17	0.85 ± 0.15	0.89 ± 0.19
TSH, μIU/mL	2.44 ± 0.94	2.42 ± 0.92	2.47 ± 0.99

BMI = body mass index; BP = blood pressure; LVDD = left ventricular diastolic dysfunction; SD = standard deviation. Continuous variables compared with the Mann-Whitney *U* test or independent *t*-test; categorical variables compared with the chi-squared or Fisher's exact test as appropriate. p < 0.05 considered statistically significant. HbA1c = glycated haemoglobin; HDL = high-density lipoprotein; LDL = low-density lipoprotein; LVDD = left ventricular diastolic dysfunction; SD = standard deviation; TSH = thyroid stimulating hormone. Continuous variables expressed as mean ± SD. Group comparisons used the Mann-Whitney *U* test or independent *t*-test (continuous) and Fisher's exact test (categorical). Lipid risk categories follow Adult Treatment Panel III thresholds. p < 0.05 considered statistically significant.

### 3.2 Prevalence of left ventricular diastolic dysfunction

**Fig. 1:**
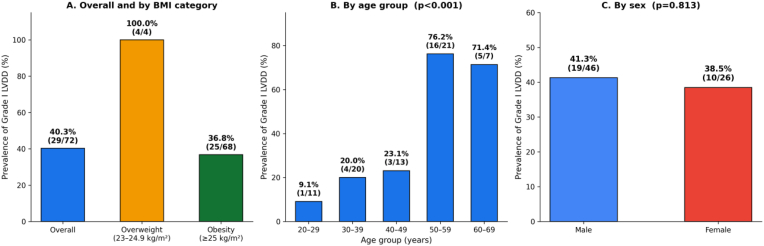
Prevalence of Grade I left ventricular diastolic dysfunction (LVDD) overall and stratified by selected variables. (A) Overall and by Asian BMI category (Fisher's exact p = 0.023 for BMI <25 vs ≥ 25 kg/m^2^). (B) By age group (Fisher's exact p < 0.001 across categories). (C) By sex (chi-squared p = 0.813). Bar values are percentages with absolute counts in parentheses. BMI = body mass index.

Grade I LVDD was identified in 29 of 72 participants (40.3%, 95% CI: 29.0–52.5%); no participant met criteria for higher-grade dysfunction. When stratified by Asian BMI categories, all 4 participants in the overweight stratum had Grade I LVDD compared with 25 of 68 (36.8%) in the obesity stratum (Fig. 1A). Although the contrast was statistically significant when analysed as BMI <25 vs ≥ 25 kg/m^2^ (Fisher's exact p = 0.023), this finding was driven by the small overweight subgroup and should be interpreted cautiously. Stratification by age group revealed a marked gradient: prevalence rose from 9.1% in adults aged 20–29 years to 76.2% in those aged 50–59 years (Fisher's exact p < 0.001; Fig. 1B). Prevalence did not differ between men (41.3%) and women (38.5%; chi-squared p = 0.813; Fig. 1C).

### 3.3 Demographic, anthropometric, and biochemical correlates

Adults with Grade I LVDD were on average 13.5 years older than those with normal diastolic function (51.5 ± 9.8 vs 37.9 ± 10.6 years; p < 0.001) and had modestly higher systolic blood pressure (124.0 ± 9.6 vs 118.5 ± 10.2 mmHg; p = 0.017). Mean BMI did not differ between groups (27.9 ± 3.1 vs 28.9 ± 4.1 kg/m^2^; p = 0.283), nor did weight or height. HbA1c, although within the non-diabetic range in both groups, was higher in adults with LVDD (5.52 ± 0.39 vs 5.27 ± 0.36%; p = 0.007). Lipid abnormalities were a consistent feature of the LVDD group: LDL cholesterol (138.2 ± 29.9 vs 110.9 ± 18.2 mg/dL; p < 0.001), total cholesterol (205.9 ± 34.8 vs 173.9 ± 20.6 mg/dL; p < 0.001), and triglycerides (134.0 ± 56.0 vs 107.0 ± 35.3 mg/dL; p = 0.026) were all significantly higher (Table 2). HDL cholesterol, haemoglobin, leukocyte count, renal function, and thyroid indices did not differ between groups.

**Table 2:** Correlation between various parameters and diastolic dysfunction.

Parameter	p value
Age	**<0.001**
Age group	**<0.001**
Sex	0.813
Height	0.906
Weight	0.484
BMI	0.283
BMI category	**0.023**
Pulse rate	0.420
Systolic BP	**0.017**
Diastolic BP	0.632
Haemoglobin	0.289
Total leukocyte count	0.884
Platelet count	0.179
HbA1c	**0.007**
HbA1c category	0.101
HDL cholesterol	0.961
LDL cholesterol	**<0.001**
LDL category	**<0.001**
Triglycerides	**0.026**
Total cholesterol	**<0.001**
Total cholesterol category	**<0.001**
Creatinine	0.389
TSH	0.818

p < 0.05 was considered statistically significant.

### 3.4 Echocardiographic findings

**Fig. 2:**
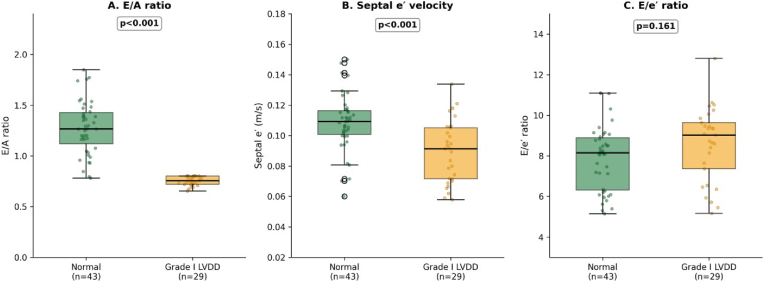
Distribution of key echocardiographic parameters by left ventricular diastolic function status. Box-and-whisker plots with overlaid individual data points showing (A) E/A ratio, (B) septal e′ velocity, and (C) E/e′ ratio in adults with normal diastolic function (n = 43) and Grade I left ventricular diastolic dysfunction (n = 29). The middle horizontal line represents the median, the upper and lower box bounds represent the 75th and 25th centiles, and the whiskers represent the Tukey limits. Group comparisons used the Mann-Whitney *U* test. E = early mitral inflow velocity; A = late (atrial) mitral inflow velocity; e′ = early diastolic mitral annular velocity; LVDD = left ventricular diastolic dysfunction.

Echocardiographic indices clearly distinguished the two groups (Table 3, Fig. 2). The E/A ratio was approximately 43% lower in adults with Grade I LVDD (0.76 ± 0.06 vs 1.33 ± 0.28; p < 0.001), and septal e′ velocity was approximately 18% lower (0.09 ± 0.02 vs 0.11 ± 0.02 m/s; p < 0.001). The E/e′ ratio trended higher in the LVDD group (8.29 ± 2.06 vs 7.61 ± 1.86; p = 0.161) but remained <14 in every participant, consistent with early disease without overt elevation of filling pressures. Peak TR velocity was marginally higher in the LVDD group (0.61 ± 0.06 v0.58 ± 0.06 m/s; p = 0.056). LVEF was preserved and indistinguishable between groups (64.6 ± 2.4 vs 64.8 ± 2.0%; p = 0.366).

**Table 3:** Echocardiographic parameters of study participants stratified by left ventricular diastolic function status.

Parameter	Overall (n = 72)	Normal diastolic function (n = 43)	Grade I LVDD (n = 29)	p value
E/A ratio	1.10 ± 0.36	1.33 ± 0.28	0.76 ± 0.06	<0.001
Septal e′ velocity, m/s	0.10 ± 0.03	0.11 ± 0.02	0.09 ± 0.02	<0.001
E/e′ ratio	7.88 ± 1.96	7.61 ± 1.86	8.29 ± 2.06	0.161
Peak TR velocity, m/s	0.59 ± 0.06	0.58 ± 0.06	0.61 ± 0.06	0.056
LV ejection fraction, %	64.7 ± 2.2	64.8 ± 2.0	64.6 ± 2.4	0.366

E = early mitral inflow velocity; A = late (atrial) mitral inflow velocity; e′ = early diastolic mitral annular velocity (tissue Doppler); LV = left ventricular; LVDD = left ventricular diastolic dysfunction; SD = standard deviation; TR = tricuspid regurgitation. Values expressed as mean ± SD. Group comparisons used the Mann-Whitney *U* test for non-normally distributed variables. p < 0.05 considered statistically significant.

### 3.5 Univariable predictors of grade I LVDD

On univariable logistic regression (Table 4), each one-year increase in age was associated with a 12.3% increase in the odds of Grade I LVDD (OR 1.12, 95% CI 1.07–1.19; p < 0.001; Nagelkerke R^2^ = 0.38). LDL cholesterol (OR 1.06 per mg/dL, 95% CI 1.03–1.09; p < 0.001) and total cholesterol (OR 1.05 per mg/dL, 95% CI 1.02–1.08; p < 0.001) were also strong predictors, as was HbA1c (OR 5.62 per percentage point, 95% CI 1.55–20.3; p = 0.008). Sex was not predictive (OR 1.13, 95% CI 0.42–3.01; p = 0.813). BMI as a continuous variable did not reach significance (OR 0.92, 95% CI 0.81–1.05; p = 0.215), although BMI ≥25 kg/m^2^ as a binary indicator was significantly associated with diastolic dysfunction in this small cohort (Fisher's exact p = 0.023). Given the modest sample size and the high collinearity among lipid and glycaemic variables, no multivariable model was fitted.

**Table 4:** Univariable logistic regression analyses of candidate predictors of Grade I left ventricular diastolic dysfunction.

Predictor	Odds ratio	95% CI	p value
Age, per 1-year increase	1.123	1.067–1.194	<0.001
Age category (50–59 vs 20–29 yr)	32.0	4.56–668.0	0.003
Sex (male vs female)	1.13	0.42–3.01	0.813
BMI, per 1-kg/m^2^ increase	0.92	0.81–1.05	0.215
BMI ≥25 vs < 25 kg/m^2^	0.13	0.02–1.00	0.040
Systolic BP, per 1-mmHg increase	1.06	1.01–1.12	0.020
HbA1c, per 1% increase	5.62	1.55–20.30	0.008
LDL cholesterol, per 1-mg/dL increase	1.06	1.03–1.09	<0.001
Total cholesterol, per 1-mg/dL increase	1.05	1.02–1.08	<0.001
Triglycerides, per 1-mg/dL increase	1.014	1.003–1.026	0.018

BMI = body mass index; BP = blood pressure; CI = confidence interval; HbA1c = glycated haemoglobin; LDL = low-density lipoprotein. Each row represents an independent univariable logistic regression model with Grade I left ventricular diastolic dysfunction as the dependent variable. No multivariable model was fitted because of the modest sample size and high collinearity among lipid and glycaemic variables. Estimates are exploratory and should be interpreted with caution.

## 4 Discussion

In this single-centre cross-sectional study of 72 adults with overweight or obesity, preserved LVEF, with no previously diagnosed cardiovascular disease or other established causes of diastolic dysfunction, we found that approximately two of every five had Grade I left ventricular diastolic dysfunction. Age, dysglycaemia in the prediabetic range, and dyslipidaemia were the strongest discriminators. Echocardiographic indices of myocardial relaxation (E/A and septal e′) were sharply reduced, whereas the E/e′ ratio trended higher but remained below the threshold for elevated filling pressures. These observations support the concept that diastolic impairment is an early, prevalent, and clinically silent manifestation of obesity-related cardiomyopathy [5,6,8,13,14,20,21].

The prevalence we observed (40.3%) sits within the range reported for adults with obesity in earlier echocardiographic studies. Russo et al. [13], in a community-based elderly cohort, demonstrated graded reductions in E/A and increases in E/e′ with rising BMI. Wang et al. [15] reported subclinical diastolic dysfunction in 42% of metabolically unhealthy adults with obesity compared with 18% of metabolically healthy controls. In Indian populations, Gade et al. [5] documented diastolic dysfunction in approximately 38% of adults with obesity, while Mamatha et al. [22] reported impaired diastolic parameters in 54% of asymptomatic Indian adults with obesity. Our findings are consistent with these data and reinforce the observation that subclinical LVDD is common in apparently healthy adults with overweight or obesity.

The strong age gradient deserves emphasis. The prevalence of Grade I LVDD rose more than eight-fold between the youngest and the 50–59 year age groups. Age-related myocardial fibrosis, endothelial dysfunction, impaired calcium reuptake, and cumulative metabolic exposure plausibly explain this pattern [13,19,23]. In South Asian populations, an early cardiometabolic ageing phenotype has been described, in which visceral adiposity, insulin resistance, and genetic susceptibility advance myocardial involvement at younger ages [7]. The implication for screening is important: the threshold for considering diastolic assessment in adults with overweight or obesity who are otherwise well may need to fall as the patient enters the fifth decade.

An additional consideration is the potential influence of menopause-related hormonal changes among older female participants, as estrogen deficiency has been associated with impaired myocardial relaxation and increased susceptibility to HFpEF; however, menopausal status was not collected in the present study and therefore could not be examined.

BMI deserves separate comment. Mean BMI did not differ between adults with and without diastolic dysfunction in our cohort, and BMI as a continuous variable did not predict LVDD on regression. This is partly explained by the narrow BMI distribution within an already overweight cohort. It is also consistent with the broader observation that BMI imperfectly captures cardiac risk in South Asian populations, where visceral adiposity, ectopic fat deposition, and metabolic perturbations operate at lower BMI thresholds [7,19,24]. The signal we observed for BMI ≥25 kg/m^2^ as a binary indicator should be interpreted cautiously because it was driven by a small overweight subgroup, all of whom had Grade I LVDD; this is most likely a reflection of an older mean age in the overweight stratum rather than a genuine inverse BMI effect.

The associations with HbA1c, LDL cholesterol, total cholesterol, and triglycerides — all within ranges that fall short of conventional disease thresholds — point to early metabolic mechanisms that converge on the myocardium. Insulin resistance, advanced glycation end-product deposition, mitochondrial dysfunction, and microvascular impairment have all been implicated in diastolic stiffening [18,19,23,25]. Patil and Burji [26] reported diastolic dysfunction in 48% of adults with HbA1c >5.7% compared with 22% in normoglycaemic counterparts, and Vadapalli and Devarakonda [27] documented reduced e′ and elevated E/e′ in adults with prediabetic glycaemic ranges. Our findings are consonant with these observations and suggest that aggressive lifestyle and pharmacologic management of even subclinical metabolic abnormalities deserves prospective evaluation.

The pattern of echocardiographic abnormalities — markedly reduced E/A and e′ but preserved E/e′ — is the signature of impaired relaxation without elevated filling pressures, which is the defining feature of Grade I LVDD per ASE/EACVI 2016 criteria [11]. Comparable values for E/A (0.75–0.85) and septal e′ (0.085–0.09 m/s) have been reported by Pascual et al. [14], Kossaify and Nicolas [16], Peterson et al. [28], and Lee et al. [18] in adults with obesity, providing reassuring external consistency. The clinical relevance lies upstream: Grade I LVDD identifies a window in which lifestyle intervention, lipid optimisation, and weight management may prevent progression. Recent randomised data with glucagon-like peptide-1 receptor agonists in HFpEF and obesity [29] reinforce the case for early detection.

The study specifically evaluated overweight and obese adults without overt cardiovascular disease, enabling the identification of early and subclinical myocardial changes related to excess body weight. Subjects with known systolic dysfunction were excluded allowing study to accurately assess the impact of obesity on left ventricular diastolic dysfunction. Use of standardized categorization of biochemical parameters improved interpretability, reproducibility and clinical applicability of findings.

## 5 Limitations

The study also has certain limitations that warrant consideration-Although adequate for identifying overall associations, the sample size limits detailed subgroup analyses. Cross-sectional nature of the study limits the ability to determine the temporal relationship between obesity and progression of diastolic dysfunction. Advanced echocardiographic markers (left atrial volume index, global longitudinal strain) were not included; these would have refined phenotyping and warrant inclusion in future protocols.

The broad age range of participants (18–65 years) introduced substantial age-related heterogeneity, which may have influenced the observed associations between age, metabolic parameters, and diastolic dysfunction. Although increasing age emerged as a strong correlate of Grade I LVDD, the cross-sectional design precludes determination of whether age acted independently or as a surrogate for cumulative metabolic exposure. Furthermore, women constituted only 36.1% of the study population, limiting the ability to evaluate sex-specific differences. Menopausal status was not recorded; therefore, the potential contribution of estrogen deficiency and menopause-related myocardial remodeling to the higher prevalence of LVDD among older participants could not be assessed. In addition, the sample size calculation was based on a previously published study conducted in a younger cohort with a different sex distribution and study design. As a result, the prevalence estimate used for sample size determination may not fully reflect the characteristics of our population, potentially limiting statistical power for subgroup analyses. Larger multicentre prospective studies with balanced representation across age groups and sexes, together with assessment of menopausal status and body fat distribution, are needed to validate these findings.

## 6 Conclusion

Grade I left ventricular diastolic dysfunction was present in 40.3% of adults with overweight or obesity and preserved ejection fraction who had no previously diagnosed cardiovascular disease or other recognised causes of diastolic dysfunction. Advancing age, higher HbA1c, and higher LDL and total cholesterol levels were associated with the presence of LVDD, whereas BMI as a continuous variable did not distinguish participants with and without dysfunction.

These findings indicate that subclinical impairment of diastolic function may be common among adults with overweight or obesity before the development of overt cardiovascular disease. The observed associations between metabolic parameters and LVDD warrant confirmation in larger prospective studies and may help inform future risk-stratification strategies aimed at understanding progression toward clinically significant cardiac dysfunction.

## 7 Key clinical takeaway messages

• Echocardiographic screening for diastolic dysfunction should be considered in adults with overweight or obesity, particularly from the fifth decade onwards, even when blood pressure, glycaemic status, and ejection fraction are within normal ranges.
• Mild metabolic perturbations within prediabetic and high-risk lipid ranges identify adults at greater risk of subclinical diastolic dysfunction, supporting early lifestyle and pharmacologic optimisation before formal disease thresholds are met.
• BMI alone may underestimate cardiac vulnerability in South Asian adults; complementary metabolic and echocardiographic assessment is needed to identify those who would benefit most from preventive interventions for heart failure with preserved ejection fraction.

## Authors contribution

PAK: Conceptualization, Methodology, Supervision, Validation, Formal analysis, Writing – review & editing.

NC: Conceptualization, Methodology, Investigation, Data curation, Formal analysis, Writing – original draft, Visualization.

HMK: Supervision, Methodology, Validation, Project administration, Writing – review & editing.

KL: Data curation, Investigation, Resources.

PAK and NC have contributed equally to this work and share first authorship.All authors have read and approved the final manuscript.

## Ethics approval and consent to participate

This study was approved by the Institutional Ethics Committee of Kasturba Medical College Manipal, Manipal Academy of Higher Education, Karnataka, India (Reference: IEC2: 205/2024). Written informed consent was obtained from all participants prior to enrolment.

## Declaration of use of artificial intelligence

During the preparation of this manuscript, the authors used artificial intelligence-assisted language tools(Claude, Anthropic) to improve grammar, readability, and language clarity. The technology was not used to generate, analyse or interpret primary data, nor to draft scientific content beyond editorial assistance. The authors have reviewed and edited all generated content and take full responsibility for the content of the final published manuscript.

## Funding

No funding was received for conducting this study.

## Competing interests

The authors declare that they have no competing interests.
